# Longitudinal Relationships Between Positive Psychological Capacities and Emotional Well-Being Among 950 College Students: A Cross-Lagged Panel Network Analysis

**DOI:** 10.3390/bs16060894

**Published:** 2026-06-02

**Authors:** Ji Dai, Xuan Xia, Dini Xue

**Affiliations:** 1 Mental Health Education Center, Hunan University of Technology and Business, Changsha 410205, China; 2076@hutb.edu.cn (J.D.); 3073@hutb.edu.cn (X.X.); 2 Institute of Developmental Psychology, Faculty of Psychology, Beijing Normal University, Beijing 100875, China

**Keywords:** positive psychological capacities, emotional well-being, college students, panel network

## Abstract

Emotional problems have become increasingly prevalent among university students, underscoring the importance of identifying protective psychological capacities that are linked to lower vulnerability to emotional problems. However, prior research has largely relied on cross-sectional designs and conventional statistical approaches, which limit the ability to clarify the temporal associations among multiple variables. To address this gap, we recruited 950 undergraduate students (61.6% female; *M*_age_ = 19.26, *SD* = 1.18) from 20 universities and conducted a two-wave longitudinal study. Cross-lagged panel network analysis was applied to examine the prospective associations linking positive psychological capacities (e.g., resilience, mindfulness) with emotional outcomes (e.g., negative affect, depression). Results revealed that positive affect and the acceptance dimension of mindfulness were among the most influential nodes within the network and exhibited stronger prospective associations with other positive psychological capacities. Based on the pathways identified in the network analysis, a half-longitudinal mediation model was further estimated to examine whether acceptance and awareness were prospectively associated with lower depressive symptoms through optimism. Together, these findings further clarified the temporal associations among positive psychological capacities and identified a prospective association linking mindfulness and depressive symptoms. These findings suggest that future mental health interventions for university students may benefit from incorporating strategies that promote positive affect and optimism within mindfulness practices.

## 1. Introduction

### 1.1. Emotional Problems Among University Students

Mental health problems among university students represent an increasingly recognized public health concern. Rates of emotional problems in this population are consistently higher than those observed in the general population (Zhang et al., 2025). Among these, depression and anxiety are the most commonly reported issues. A recent meta-analysis estimated global prevalence rates of 33.6% for depressive symptoms and 39.0% for anxiety symptoms in university students (W. Li et al., 2022). In China, these rates are similarly elevated, with depression and anxiety reported at 34.7% and 25.0%, respectively (Lin et al., 2025; X. Wang & Liu, 2022). Affective well-being, including both positive and negative affect, represents a core dimension of psychological health in young adults. Negative affect has consistently been associated with broader psychological distress (Stanton & Watson, 2014). In contrast, diminished positive affect has been identified as a transdiagnostic feature of mental disorders, such as major depressive disorder and schizophrenia (Guineau et al., 2023; Watson & Naragon-Gainey, 2010). Collectively, these patterns underscore the importance of identifying protective factors that can buffer against emotional vulnerability and support psychological adjustment during the university years.

### 1.2. Positive Psychological Capacities Reducing Emotional Problems

Research has increasingly shifted from an exclusive emphasis on risk factors toward understanding the role of positive psychological capacities in psychological adjustment and mental health. Among these capacities, emotion regulation, mindfulness, and resilience have received considerable attention. Within the emotion regulation literature, cognitive reappraisal (CR) and expressive suppression (ES) are among the most widely studied strategies. Cognitive reappraisal, an antecedent-focused strategy, involves reframing the meaning of a situation before an emotional response is fully generated. Greater use of CR has been consistently associated with greater positive affect and higher well-being (Dawel et al., 2024; Southward et al., 2022). Expressive suppression, a response-focused strategy, refers to the deliberate inhibition of ongoing emotional expression (e.g., facial, vocal, or gestural displays) after an emotional response has already been fully elicited. Higher levels of expressive suppression have been associated with lower positive affect, elevated depressive and anxiety symptoms, and increased suicidal ideation (Fernandes & Tone, 2021; Forkmann et al., 2014). Mindfulness represents another positive psychological capacity closely associated with emotional functioning. Mindfulness refers to present-centered awareness of internal (e.g., bodily sensations) and external contexts, maintained with an attitude of acceptance and equanimity (Chems-Maarif et al., 2025). Accumulating evidence suggests that mindfulness is associated with more adaptive emotional outcomes among university students. For example, a brief three-day mindfulness intervention has been associated with reductions in anxiety symptoms (Sousa et al., 2021), and online mindfulness-based interventions have also been found to alleviate depressive symptoms in this population (Barcaccia et al., 2024). Resilience is also a key positive psychological capacity, particularly relevant during the university years, which are often marked by a range of stressors (Hurst et al., 2013). Defined as the capacity to maintain or restore psychological well-being in the face of adversity (Herrman et al., 2011), resilience buffers the negative effects of stress on mental health. Individuals with higher levels of resilience typically rely on active, problem-focused coping strategies to manage stress (Ulibarri-Ochoa et al., 2024), whereas those with lower resilience are more prone to depression and anxiety under stressful conditions (Anyan & Hjemdal, 2016).

### 1.3. The Interrelation Among Positive Psychological Capacities

These three constructs do not operate in isolation but form a dynamic system of interacting associations, as reflected in a growing body of empirical work documenting their complex interrelations. Both mindfulness and emotion regulation have been independently linked to resilience, and the mindfulness stress-buffering account (Creswell & Lindsay, 2014) and affect-regulation framework of resilience (Troy et al., 2023) posit that mindfulness and adaptive emotion regulation contribute to resilience by strengthening individuals’ capacity to tolerate and recover from stress. Empirical findings further suggest that mindfulness may facilitate emotion regulation, with acceptance and awareness being associated with lower emotional reactivity at both subjective and neural levels (Chambers et al., 2009; Grecucci et al., 2015). Furthermore, resilience itself may condition how individuals engage with mindfulness: those with more resilient profiles have been found to derive greater benefit from mindfulness-based interventions relative to those with more vulnerable profiles (Godara & Singer, 2024). At the level of indirect effects, empirical studies have demonstrated that mindfulness may indirectly facilitate resilience by alleviating difficulties in emotion regulation, and it has also been identified as a mediator linking emotion regulation and resilience (Arpacı & Tanrıverdi, 2024; T. Li et al., 2025; Zarotti et al., 2020). These interrelations suggest that mindfulness, emotion regulation, and resilience may function as components of a broader network of associations rather than as isolated predictors.

### 1.4. The Current Study

Although prior research has suggested that emotion regulation, resilience, and mindfulness may be reciprocally related, their interrelations have often not been examined simultaneously within a broader psychological system. Moreover, when these constructs are examined separately, estimates of their associations with emotional outcomes may be incomplete, as the observed effect of one capacity may partly reflect its interplay with other psychological resources. To address these limitations, the present study applies a cross-lagged panel network (CLPN), which conceptualizes psychological constructs as interconnected systems and estimates directed temporal associations among multiple variables after controlling for autoregressive stability and within-wave associations (Wysocki et al., 2025). The study further extends prior work by examining these capacities at a more fine-grained level, allowing their distinct components to be examined as prospectively interconnected over time.

Using CLPN, the present study aims to (1) examine the temporal associations among dimensions of positive psychological capacities and emotional outcomes, as well as identify influential nodes within the network, and (2) identify prospective associations linking positive psychological capacities to emotional outcomes within the network and further evaluate these associations using half-longitudinal mediation model. These findings may help identify psychological capacities that represent promising intervention targets for promoting emotional health among university students. Based on prior evidence, mindfulness, adaptive emotion regulation and resilience are expected to be associated with emotional health within the CLPN while accounting for bidirectional effects among variables. Network analyses are treated as exploratory in identifying pathways and central nodes, whereas SEM analyses are confirmatory without a clear hypothesis.

## 2. Materials and Methods

### 2.1. Participants and Procedure

This study employed a two-wave longitudinal design. Participants were recruited via cluster random sampling from more than 20 universities across Hunan Province (Jishou, Changsha), Shanghai, and Hainan Province (Haikou, Sanya), China. Participants who provided contact information and consented to follow-up were contacted for the second wave. The sample was drawn from a relatively socioeconomically disadvantaged population. Specifically, SES was derived using principal component analysis based on multiple indicators. Individual SES scores were computed and ranked, and participants in the lowest 14% of the distribution were selected, representing relatively disadvantaged individuals. At baseline, 1159 undergraduate students completed the survey (61.6% female, 38.4% male; age range = 17–24 years, *M* = 19.26, *SD* = 1.18). Two years later, 950 of these students participated in the follow-up survey (61% female, 39% male; age range = 17–23 years, *M* = 19.23, *SD* = 1.18), yielding an overall retention rate of 82.0%. T tests assessing attrition revealed that participants who dropped out did not differ significantly from those who were retained on demographic variables (e.g., age and SES) or the psychological variables assessed in the present study (see Supplementary Table S3). In addition, a chi-square test showed no significant difference in gender distribution between the two groups, *χ*^2^ (1, *n* = 1131) = 0.809, *p* = 0.369. Prior to data collection, participants were provided with a detailed explanation of the study’s objectives and procedures. They were informed that their participation was entirely voluntary, and that they could discontinue at any stage without any negative consequences.

### 2.2. Measures

#### 2.2.1. Self-Report Demographic Data Questionnaire

Socio-demographic data were collected using a self-report questionnaire, which included participants’ gender, age, residential area, and subjective socioeconomic status (SES). The SES section assessed participants’ parental educational attainment, parental occupation, and monthly household income. Participants were also asked to evaluate their family’s relative socioeconomic standing within their province (ranging from 1 = lowest to 10 = highest) and their relative position among classmates at school (ranging from 1 = lowest to 10 = highest). A composite SES score was calculated by standardizing all indicators and extracting the principal component through principal component analysis.

#### 2.2.2. Resilience

Resilience was assessed using Chinese version of Connor–Davidson Resilience Scale (CD-RISC; Connor & Davidson, 2003), translated and culturally adapted by Yu and Zhang (2007). The original five-factor structure was revised to a three-factor model to better reflect the Chinese cultural context. The three subscales are optimism (4 items; tendency to view situations positively and trust in personal and social resources), strength (8 items; ability to recover from setbacks and even gain energy and motivation following challenging experiences), and tenacity (13 items; equanimity, promptness, perseverance, and sense of control in the face of adversity). Sample items from each subscale are “I see the humorous side of things”, “I tend to bounce back after illness or hardship”, “I like challenges”, respectively. Each item was rated on a 5-point Likert scale ranging from 1 (never) to 5 (always). In the present study, separate total scores were calculated for each subscale to assess participants’ levels of optimism, strength, and tenacity, with higher scores indicating greater resilience in the respective domain. In the current study, the Cronbach’s α coefficients for the optimism subscale were 0.633 at T1 and 0.641 at T2, for the strength subscale were 0.839 at T1 and 0.866 at T2, and for the tenacity subscale were 0.905 at T1 and T2.

#### 2.2.3. Emotion Regulation

Emotion regulation was assessed using the Chinese version of the Emotion Regulation Questionnaire (ERQ; Gross & John, 2003), which was translated and validated by L. Wang et al. (2007). The questionnaire measures how individuals manage and control their emotional experiences and consists of two subscales: cognitive reappraisal and expressive suppression. The cognitive reappraisal subscale included 6 items (e.g., “When I want to feel more positive emotion (such as joy or amusement), I change what I’m thinking about.”). The expressive suppression subscale included 4 items (e.g., “When I am feeling positive emotions, I am careful not to express them.”). All items used a 7-point Likert scale, ranging from 1 (strongly disagree) to 7 (strongly agree). Separate total scores were calculated for each subscale, with higher scores representing a greater frequency of using that specific emotion regulation strategy. In the current study, the Cronbach’s α coefficients for the cognitive reappraisal subscale were 0.848 at T1 and 0.864 at T2, and for the expression suppression subscale were 0.703 at T1 and 0.742 at T2.

#### 2.2.4. Mindfulness

Mindfulness was assessed by the Chinese version of the Freiburg Mindfulness Inventory (FMI; Walach et al., 2006), as translated and adapted by Chen and Zhou (2014), with the original 14-item scale reduced to 12 items to enhance its applicability to Chinese non-clinical populations. The scale comprises two subscales: awareness and acceptance. The Awareness subscale measured participants’ sustained monitoring of current internal and external stimuli, consisting of 5 items (e.g., “I am open to the experience of the present moment”). The Acceptance subscale measured participants’ non-judgmental and non-reactive attitude toward their experiences, consisting of 7 items (e.g., “I am able to appreciate myself”). All items used a 4-point Likert scale from 1 (rarely) to 4 (often). A total score was calculated for each subscale, with higher scores indicating greater levels of awareness and acceptance. In the current study, the Cronbach’s α coefficients for the awareness subscale were 0.747 at T1 and 0.794 at T2, and for the acceptance subscale were 0.811 at T1 and 0.860 at T2.

#### 2.2.5. Depression and Anxiety

Depression and anxiety were measured using the corresponding subscales of the Chinese version of the Brief Symptom of Inventory (BSI; Derogatis, 2001), as translated by M. Li et al. (2018). The full inventory encompasses a four-factor structure, comprising somatization, depression, anxiety, and panic (Andreu et al., 2008). In the present study, only the depression and anxiety subscales were administered to assess participants’ levels of depressive and anxiety symptoms. The depression subscale consisted of 6 items, such as “Feeling no interest in things”. The anxiety subscale consisted of 3 items, such as “Nervousness or shakiness inside”. Participants indicated the extent to which they had experienced depressive and anxiety symptoms during the previous week on a 5-point Likert scale ranging from 1 (not at all) to 5 (extremely). For each subscale, a total score was calculated, with higher scores indicating greater severity of depression or anxiety. In this study, the Cronbach’s α coefficients for the depression subscale were 0.823 at T1 and 0.866 at T2, and for the anxiety subscale were 0.762 at T1 and 0.788 at T2.

#### 2.2.6. Positive and Negative Affect

Positive and negative affect were assessed with the Chinese version of The Scale of Positive and Negative Experience (SPANE; Jovanović et al., 2020), as translated by F. Li et al. (2013). The SPANE is designed to capture positive and negative emotional experiences regardless of their sources, arousal level, or cultural context. The instrument comprises two subscales, each containing six items that measure positive affect (PA; e.g., “Happy”) and negative affect (NA; e.g., “Unpleasant”), respectively. Participants rated how frequently they had experienced PA and NA over the past week on a 5-point Likert scale ranging from 1 (very rarely) to 5 (very often). For each subscale, total scores were calculated, with higher scores reflecting greater positive affect or negative affect. In the current study, the Cronbach’s α coefficients for the PA subscale were 0.908 at T1 and 0.916 at T2, and for the NA subscale were 0.857 at T1 and 0.869 at T2.

### 2.3. Statistical Analyses

#### 2.3.1. Network Analysis

Descriptive statistics and correlations were computed in JASP (Version 0.95.1). CLPN was employed to examine the temporal associations among variables across two waves (T1, T2). This method accounts for the autoregressive effects of each node while simultaneously modeling both intra- and inter-correlations across time, thereby estimating the extent to which a variable at one occasion predicts other variables at the subsequent time point. Model estimation was performed in R (Version 4.5.1) using the glmnet package (Friedman et al., 2010), with Gaussian LASSO regularization and 10-fold cross-validation applied to promote network sparsity and mitigate overfitting. The directed networks were visualized by qgraph package (Epskamp et al., 2012).

Beyond network structure, we calculated centrality indices to evaluate the relative importance of nodes: in-expected influence (in-EI) and out-expected influence (out-EI; Niu et al., 2023). The in-EI index measured the proportion of variance in a node that was accounted for by other nodes at the prior time point, with higher in-EI values indicating greater susceptibility to preceding influences. Conversely, out-EI captures the extent to which a node predicts other nodes at the next time, with higher out-prediction values denoting stronger prospective influence on subsequent variables.

#### 2.3.2. Network Accuracy and Stability

To evaluate the robustness of our network findings, we employed bootstrapping routines implemented in the bootnet package in R (Epskamp et al., 2018). A nonparametric bootstrap procedure with 1000 bootstrap samples was conducted to generate 95% confidence intervals (CIs) around edge weights. Narrower CIs indicate higher accuracy in estimating edge parameters, whereas wide intervals signal potential instability. In addition, we applied the case-dropping subset bootstrap to examine the stability of centrality metrics. This procedure yields the correlation stability (CS) coefficient, which reflects the maximum proportion of cases that can be randomly dropped while retaining a correlation of at least 0.70 between centrality indices computed from the full dataset and those estimated from the subsets. A CS coefficient above 0.25 is generally considered acceptable, and values above 0.50 provide strong evidence of robustness (Epskamp et al., 2018).

#### 2.3.3. Half-Longitudinal Mediation Model

Building on the path results obtained from the CLPN, we further conducted cross-lagged structural equation modeling to validate these findings. Following the guidelines proposed by Fang et al. (2021) and previous research (Deniz et al., 2023), in a half-longitudinal mediation model, the mediation pathway can be evaluated by testing two critical paths: X1 → M2 and M1 → Y2, where the initial variable at Time 1 (X1) predicts the mediator at Time 2 (M2), and, separately, the mediator at Time 1 (M1) predicts the outcome variable at Time 2 (Y2).

## 3. Results

### 3.1. Network Stability and Accuracy

The descriptive statistics and correlation results at T1 and T2 are presented in Supplementary Table S1. The 95% bootstrapped confidence intervals (CIs) of edge weights showed moderate intervals (see Supplementary Figure S1), which revealed that the stability of the network was acceptable. The case-dropping bootstrap procedure revealed CS-coefficients of 0.36 for in-EI and 0.21 for out-EI (see Supplementary Figure S2), indicating the stability of in-EI was acceptable (>0.25) and that findings based on out-EI (<0.25) should be interpreted with caution. Results of the edge-weight difference tests and centrality difference tests are displayed in Supplementary Figures S3 and S4.

### 3.2. Cross-Lagged Panel Network Structure

**Figure 1:**
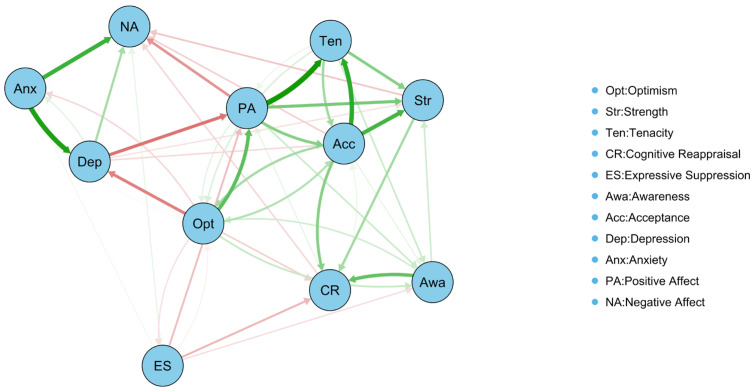
Cross-Lagged panel network. Arrows represent longitudinal relationships. Green edges indicate positive relationships, and red edges indicate negative relationships. Thicker edges represent stronger relations.

The estimated CLPN is illustrated in Figure 1. The full adjacency matrices of all edge weights are reported in Supplementary Table S2, and the CLPN including autoregressive paths is depicted in Supplementary Figure S5. In the visualization, green arrows represent positive longitudinal associations, red arrows represent negative associations, and arrow thickness reflects the strength of unique longitudinal relationships. The CLPN result showed that of 110 possible edges, 50 are not zero after regularization convergence and omission of autoregressive paths. In T1 → T2 network, regularized cross-lagged associations showed low overall effect sizes, though several paths exhibited greater relative weights. Specifically, node “PA” (positive affect) showed a cross-lagged edge to node “Ten” (tenacity; weight = 0.153); node “Anx” (anxiety) showed a cross-lagged edge to node “Dep” (depression; weight = 0.140); and node “Acc” (acceptance) showed a cross-lagged edge to node “Ten” (tenacity; weight = 0.128).

The positive psychological capacities variables clustered together. Moreover, the network structure revealed that positive affect was associated with multiple positive psychological capacities. Specifically, positive affect showed cross-lagged associations with mindfulness acceptance, strength, and tenacity. Among emotion regulation strategies, cognitive reappraisal was associated with subsequent levels of mindfulness acceptance and awareness, whereas expressive suppression was located at the periphery of the network and was negatively associated with positive psychological capacities.

When examining the longitudinal associations between positive psychological capacities and emotional health, the results indicated that among the positive psychological variables, only positive affect was associated with lower depression at follow-up (weight = −0.077). Furthermore, optimism was associated with baseline levels of awareness and acceptance (weight_1_ = 0.055, weight_2_ = 0.029). These findings suggest a potential indirect pathway in which mindfulness facets were associated with optimism, which in turn was linked to lower levels of depression at follow-up. To examine this pathway further, we constructed a half-longitudinal mediation model.

### 3.3. Centrality Indexes

**Figure 2:**
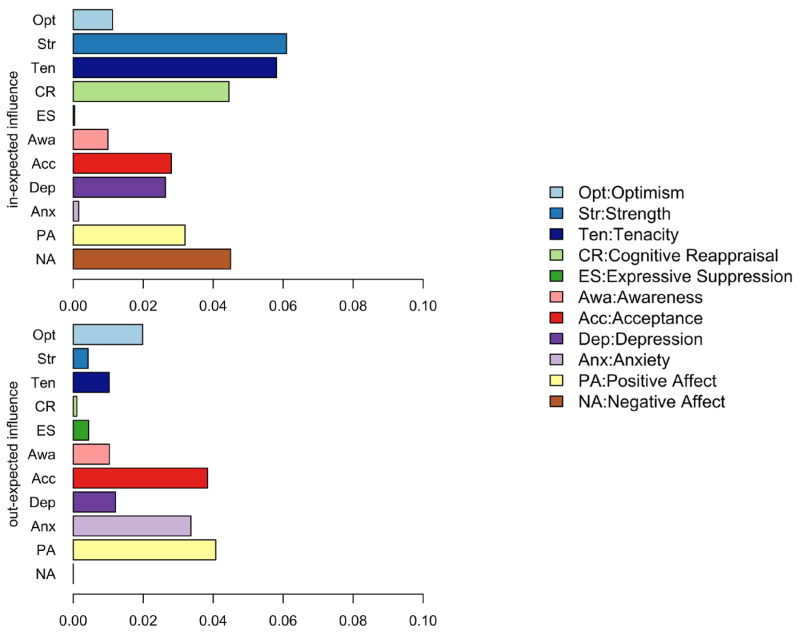
Centrality indexes for cross-lagged panel network.

The centrality indices are depicted in Figure 2 and all centrality indexes for the network nodes are presented in Table 1. For in-expected influence (in-EI), “Str” (strength, in-EI = 0.061), “Ten” (Tenacity, in-EI = 0.058), “CR” (Cognitive reappraisal, in-EI = 0.045) and “NA” (Negative affect, in-EI = 0.045) exhibited the highest values, indicating that these nodes were persistently and strongly predictable from other nodes over time. For out-expected influence (out-EI), “PA” (Positive affect, out-EI = 0.041), “Acc” (Acceptance, out-EI = 0.038) and “Anx” (Anxiety, out-EI = 0.034) showed relatively higher values.

### 3.4. Half-Longitudinal Mediation Model

**Figure 3:**
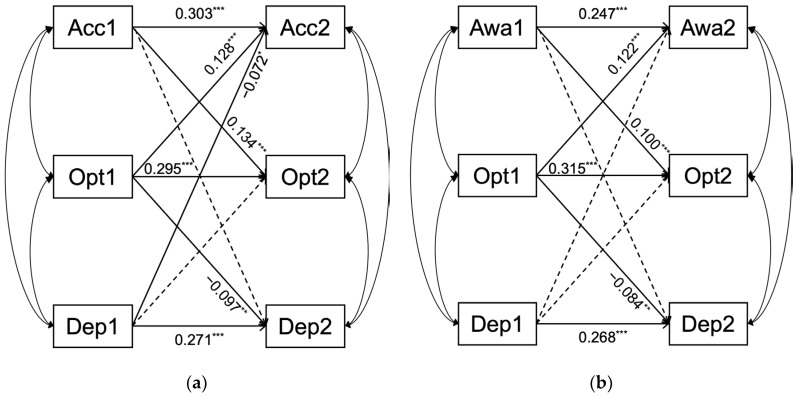
The half-longitudinal mediation model. * *p* < 0.05, ** *p* < 0.01, *** *p* < 0.001. Standardized path coefficients are presented. Solid arrows indicate statistically significant paths, and dashed arrows indicate nonsignificant paths. Schemes follow another format. (**a**) Model 1; (**b**) Model 2. Acc1: acceptance at T1, Acc2: acceptance at T2; Awa1: awareness at T1, Awa2: awareness at T2; Opt1: optimism at T1, Opt2: optimism at T2; Dep1: depression at T1, Dep2: depression at T2. The numbers in the figure are the standardized regression coefficients for the path. The solid line indicates that the regression coefficients are significant.

To further examine the potential directional associations from mindfulness to depression identified in the network analysis, we constructed a half-longitudinal mediation model to test whether optimism acted as a mediator in the longitudinal relationship between mindfulness and depression. The structural equation modeling results are presented in Figure 3. In Model 1, T1 acceptance was positively associated with optimism at follow-up, controlling for baseline levels (*β* = 0.134, *SE* = 0.037, 95%*CI* [0.044, 0.149]), and T1 optimism was significantly associated with T2 depression, controlling for baseline level of depression (*β* = −0.097, *SE* = 0.040, 95%*CI* [−0.236, −0.023]). In Model 2, T1 awareness was positively associated with T2 optimism, controlling for baseline level of optimism (*β* = 0.100, *SE* = 0.034, 95%*CI* [0.033, 0.164]), and T1 optimism showed significant association with depression at follow-up, controlling for baseline levels (*β* = −0.084, *SE* = 0.036, 95%*CI* [−0.208, −0.017]). These findings provide additional support for the proposed pathway in which the acceptance and awareness facets of mindfulness were associated with depressive symptoms through optimism over time. This pattern is consistent with a potential directional association, although mediation inference remains limited in a two-wave design.

## 4. Discussion

The network results identified positive affect and acceptance as relatively central nodes within the network and indicated a temporal association between mindfulness and depression. This association was further examined using a half-longitudinal mediation model, which provided evidence for the mediating role of optimism between mindfulness and subsequent depression. These findings provide an integrated account of how positive psychological capacities are associated with changes in emotional health over time.

Among the network results, positive affect showed relatively higher out-EI values, suggesting that it may be more strongly connected to changes in other variables over time, particularly with respect to multiple positive psychological capacities. However, given the modest stability of the centrality estimates, interpretations regarding the relative influence of this node should be made cautiously. Positive affect was prospectively associated with subsequent increases in the tenacity and strength dimensions of resilience. This pattern is broadly consistent with the broaden-and-build theory (Fredrickson, 2001), which posits that positive emotions expand individuals’ thought–action repertoires and facilitate the accumulation of psychological resources, including resilience (Luthans et al., 2007). Positive affect was also prospectively associated with the acceptance and awareness components of mindfulness. Contrary to expectations, the temporal association appeared to operate primarily from positive affect to mindfulness rather than in the opposite direction. One possible explanation is that mindfulness practices may be more closely associated with calmness and emotional stability than with heightened positive affect (Kiken et al., 2017; Lalot et al., 2014), whereas positive affect may broaden attentional scope and support adaptive cognitive processing (Paul et al., 2021), potentially facilitating mindfulness-related processes.

Acceptance also showed relatively higher out-EI values, although the limited stability of this centrality estimate suggests that its relative influence should be interpreted cautiously. Nevertheless, acceptance showed relatively consistent cross-lagged associations with multiple resilience dimensions, suggesting a close longitudinal relationship between these constructs. Associations were also observed in the opposite direction, with resilience dimensions predicting subsequent increases in acceptance, indicating that resilience and acceptance may reinforce one another over time. Both acceptance and awareness were prospectively associated with cognitive reappraisal, consistent with prior work suggesting that mindfulness-related processes are linked to emotion regulation (Chambers et al., 2009; Grecucci et al., 2015). Within the mindfulness-to-meaning framework (Garland et al., 2015), acceptance may foster a decentered metacognitive stance that allows individuals to observe internal experiences with greater psychological distance and cognitive flexibility, thereby facilitating more constructive appraisals (Z. Li et al., 2025). In addition, awareness may support reappraisal by enriching the contextual information available for cognitive processing.

The network results also suggested a potential temporal association from mindfulness to depression at follow-up. In line with the two-wave model results, baseline mindfulness facets were associated with subsequent depressive symptoms through their associations with optimism. While prior research has emphasized the protective roles of resilience and mindfulness, the present findings further suggest that mindfulness may relate to lower depressive symptoms partly through more positive future expectancies. This pattern aligns with cognitive models of depression, which highlight negative expectations about the future as a core feature contributing to its onset and maintenance (Rief & Joormann, 2019). Supporting this account, optimism, defined as a generalized positive expectancy regarding the future, has been associated with lower depression (Scheier & Carver, 1985; Kube et al., 2018). Prior research further suggests that trait mindfulness prospectively predicts optimism, and that mindfulness-based interventions may promote more optimistic perspectives (Heckenberg et al., 2019). Together, these findings suggest that optimism may partly explain the cross-lagged association between mindfulness and subsequent depressive symptoms. Finally, the network analyses also suggested associations in the opposite direction, indicating that higher baseline depressive symptoms were associated with subsequent reductions in positive affect, resilience, and mindfulness. These findings suggest that psychological resources and emotional problems may be reciprocally associated over time. Alternatively, these associations may also partly reflect stable individual differences that were not fully captured in the current models.

This study contributes to existing research by examining positive psychological capacities as an interconnected system unfolding over time, rather than as isolated factors. By applying CLPN, the present study complements traditional variable-centered approaches and provides a more fine-grained view of how positive psychological capacities may be temporally associated with one another and with emotional health, although the observed effect sizes were modest. In particular, the findings identified a potentially meaningful prospective association linking mindfulness, optimism, and depressive symptoms, which may have implications for future research and intervention development among socioeconomically disadvantaged university students.

Several limitations should be acknowledged. First, although the longitudinal design allowed us to examine temporal ordering, the two-wave structure limits stronger inferences regarding mediation. Second, while regularization improves model parsimony, it may also shrink small effects and exclude weaker yet potentially meaningful associations. Moreover, centrality indices, particularly out-EI, should be interpreted in light of their stability, and replication in independent samples is needed. Third, reliance on self-report measures may introduce recall bias and shared method variance; future studies incorporating behavioral or physiological indicators could improve measurement precision. Fourth, generalizability may be limited by the study context and sample characteristics, as participants were recruited from geographically distant cities and were socioeconomically disadvantaged. Finally, this study focused primarily on internal positive psychological capacities. Future research should also consider external protective factors, such as social connectedness, to provide a more comprehensive understanding of emotional health and resilience among university students.

## 5. Conclusions

The present study constructed a cross-lagged longitudinal network integrating positive psychological capacities and emotional health, providing a more fine-grained understanding of their temporal interrelations. Positive affect and acceptance emerged as relatively influential nodes: positive affect was associated with subsequent mindfulness and resilience, and acceptance was linked to multiple dimensions of resilience and cognitive reappraisal at follow-up. However, given the modest effect sizes and the limited stability of certain centrality indices, particularly out-EI, these findings should be interpreted with caution and replicated in future research. In addition, converging evidence from the network analysis and half-longitudinal mediation model suggested that mindfulness was prospectively associated with lower depressive symptoms through optimism.

These findings offer empirical support for interventions aimed at strengthening positive psychological capacities and addressing depressive symptoms among socioeconomically disadvantaged university students. Specifically, interventions may benefit from incorporating components that promote positive emotional experiences, such as engagement in meaningful activities and the cultivation of daily positive experiences. In addition, greater emphasis could be placed on mindfulness-based practices that foster acceptance, which may support the development of resilience and adaptive emotion regulation. Moreover, the observed temporal associations among mindfulness, optimism, and depression suggest that mindfulness-based programs incorporating techniques to foster positive future expectancies, such as best possible self-visualization, may represent a promising direction for addressing depressive symptoms among university students. Future research could extend these findings using multi-wave longitudinal designs to better capture temporal dynamics and strengthen inferences regarding directional pathways. In addition, integrating within-person modeling approaches, such as dynamic structural equation modeling, may help disentangle stable individual differences from time-varying processes.

## Figures and Tables

**Table 1:** Centrality Index Results of CLPN.

Node	In-Expected Influence	Out-Expected Influence
Optimism	0.011	0.020
Strength	0.061	0.004
Tenacity	0.058	0.010
Cognitive Reappraisal	0.045	0.001
Expressive Suppression	0.000	0.004
Awareness	0.010	0.010
Acceptance	0.028	0.038
Depression	0.026	0.012
Anxiety	0.002	0.034
Positive Affect	0.032	0.041
Negative Affect	0.045	0.000

## Data Availability

The datasets analyzed during the current study are available from the corresponding author on reasonable request.

## Supplementary Materials

The following supporting information can be downloaded at: https://www.mdpi.com/article/10.3390/bs16060894/s1, Table S1: Description and Correlation matrix; Table S2: Edge Weight Adjacency Matrix of CLPN; Table S3: Baseline Differences Between Completers and Dropouts; Figure S1: Bootstrapped 95% Confidence Intervals of the Edge Weights for CLPN; Figure S2: Case-Dropping Bootstrapped Centrality Indexes for CLPN; Figure S3: Differences in Edge Weight in CLPN; Figure S4: Differences in Centrality Indexes in CLPN; Figure S5: Cross-Lagged Panel Network with Autoregression Paths.

## Abbreviations

The following abbreviations are used in this manuscript:

Opt	Optimism
Str	Strength
Ten	Tenacity
CR	Cognitive Reappraisal
ES	Expressive Suppression
Awa	Awareness
Acc	Acceptance
Dep	Depression
Anx	Anxiety
PA	Positive Affect
NA	Negative Affect

## References

[B1-behavsci-16-00894] Andreu Y., Galdón M. J., Dura E., Ferrando M., Murgui S., García A., Ibáñez E. (2008). Psychometric properties of the Brief Symptoms Inventory-18 (Bsi-18) in a Spanish sample of outpatients with psychiatric disorders. Psicothema.

[B2-behavsci-16-00894] Anyan F., Hjemdal O. (2016). Adolescent stress and symptoms of anxiety and depression: Resilience explains and differentiates the relationships. Journal of Affective Disorders.

[B3-behavsci-16-00894] Arpacı R., Tanrıverdi D. (2024). The mediating role of emotion regulation difficulties in the correlation between mindfulness and psychological resilience in patients diagnosed with depression. The Journal of Nervous and Mental Disease.

[B4-behavsci-16-00894] Barcaccia B., Medvedev O. N., Pallini S., Mastandrea S., Fagioli S. (2024). Examining mental health benefits of a brief online mindful-ness intervention: A randomised controlled Trial. Mindfulness.

[B5-behavsci-16-00894] Chambers R., Gullone E., Allen N. B. (2009). Mindful emotion regulation: An integrative review. Clinical Psychology Review.

[B6-behavsci-16-00894] Chems-Maarif R., Cavanagh K., Baer R., Gu J., Strauss C. (2025). Defining mindfulness: A review of existing definitions and suggested refinements. Mindfulness.

[B7-behavsci-16-00894] Chen S., Zhou R. (2014). Validation of a Chinese version of the Freiburg Mindfulness Inventory–Short version. Mindfulness.

[B8-behavsci-16-00894] Connor K. M., Davidson J. R. T. (2003). Development of a new resilience scale: The Connor-Davidson Resilience Scale (CD-RISC). Depression and Anxiety.

[B9-behavsci-16-00894] Creswell J. D., Lindsay E. K. (2014). How does mindfulness training affect health? A mindfulness stress buffering account. Current Directions in Psychological Science.

[B10-behavsci-16-00894] Dawel A., Mewton P., Gulliver A., Farrer L. M., Calear A. L., Newman E., Cherbuin N. (2024). For whom and what does cognitive reappraisal help? A prospective study. Cognitive Therapy and Research.

[B11-behavsci-16-00894] Deniz M. E., Satici S. A., Okur S., Satici B. (2023). Relations among self-control, hope, and psychological adjustment: A two-wave longitudinal mediation study. Scandinavian Journal of Psychology.

[B12-behavsci-16-00894] Derogatis L. R. (2001). Brief symptom inventory 18.

[B13-behavsci-16-00894] Epskamp S., Borsboom D., Fried E. I. (2018). Estimating psychological networks and their accuracy: A tutorial paper. Behavior Research Methods.

[B14-behavsci-16-00894] Epskamp S., Cramer A. O. J., Waldorp L. J., Schmittmann V. D., Borsboom D. (2012). qgraph: Network visualizations of relationships in psychometric data. Journal of Statistical Software.

[B15-behavsci-16-00894] Fang J., Wen Z., Chiou H. (2021). Mediation analysis of longitudinal data. Journal of Psychological Science.

[B16-behavsci-16-00894] Fernandes M. A., Tone E. B. (2021). A systematic review and meta-analysis of the association between expressive suppression and positive affect. Clinical Psychology Review.

[B17-behavsci-16-00894] Forkmann T., Scherer A., Böcker M., Pawelzik M., Gauggel S., Glaesmer H. (2014). The relation of cognitive reappraisal and expressive suppression to suicidal ideation and suicidal desire. Suicide and Life-Threatening Behavior.

[B18-behavsci-16-00894] Fredrickson B. L. (2001). The role of positive emotions in positive psychology: The broaden-and-build theory of positive emotions. American Psychologist.

[B19-behavsci-16-00894] Friedman J., Hastie T., Tibshirani R. (2010). Regularization paths for generalized linear models via coordinate descent. Journal of Statistical Software.

[B20-behavsci-16-00894] Garland E. L., Farb N. A., Goldin P., Fredrickson B. L. (2015). Mindfulness broadens awareness and builds eudaimonic meaning: A process model of mindful positive emotion regulation. Psychological Inquiry.

[B21-behavsci-16-00894] Godara M., Singer T. (2024). Resilient stress reactivity profiles predict mental health gains from online contemplative training: A randomized clinical trial. Journal of Personalized Medicine.

[B22-behavsci-16-00894] Grecucci A., Pappaianni E., Siugzdaite R., Theuninck A., Job R. (2015). Mindful emotion regulation: Exploring the neurocognitive mechanisms behind mindfulness. BioMed Research International.

[B23-behavsci-16-00894] Gross J. J., John O. P. (2003). Individual differences in two emotion regulation processes: Implications for affect, relationships, and well-being. Journal of Personality and Social Psychology.

[B24-behavsci-16-00894] Guineau M. G., Ikani N., Rinck M., Collard R. M., van Eijndhoven P., Tendolkar I., Schene A. H., Becker E. S., Vrijsen J. N. (2023). Anhedonia as a transdiagnostic symptom across psychological disorders: A network approach. Psychological Medicine.

[B25-behavsci-16-00894] Heckenberg R. A., Hale M. W., Kent S., Wright B. J. (2019). An online mindfulness-based program is effective in improving affect, over-commitment, optimism and mucosal immunity. Physiology & Behavior.

[B26-behavsci-16-00894] Herrman H., Stewart D. E., Diaz-Granados N., Berger E. L., Jackson B., Yuen T. (2011). What is resilience?. The Canadian Journal of Psychiatry.

[B27-behavsci-16-00894] Hurst C. S., Baranik L. E., Daniel F. (2013). College student stressors: A review of the qualitative research. Stress and Health.

[B28-behavsci-16-00894] Jovanović V., Lazić M., Gavrilov-Jerković V., Molenaar D. (2020). The scale of positive and negative experience (SPANE). European Journal of Psychological Assessment.

[B29-behavsci-16-00894] Kiken L. G., Lundberg K. B., Fredrickson B. L. (2017). Being present and enjoying it: Dispositional mindfulness and savoring the moment are distinct, interactive predictors of positive emotions and psychological health. Mindfulness.

[B30-behavsci-16-00894] Kube T., Siebers V. H. A., Herzog P., Glombiewski J. A., Doering B. K., Rief W. (2018). Integrating situation-specific dysfunctional expectations and dispositional optimism into the cognitive model of depression—A path-analytic approach. Journal of Affective Disorders.

[B31-behavsci-16-00894] Lalot F., Delplanque S., Sander D. (2014). Mindful regulation of positive emotions: A comparison with reappraisal and expressive suppression. Frontiers in Psychology.

[B32-behavsci-16-00894] Li F., Bai X., Wang Y. (2013). The scale of positive and negative experience (SPANE): Psychometric properties and normative data in a large Chinese sample. PLoS ONE.

[B33-behavsci-16-00894] Li M., Wang M.-C., Shou Y., Zhong C., Ren F., Zhang X., Yang W. (2018). Psychometric properties and measurement invariance of the brief symptom inventory-18 among chinese insurance employees. Frontiers in Psychology.

[B34-behavsci-16-00894] Li T., Zhou D., Zhang W., Ju C. (2025). The relationship between mindfulness and test anxiety among high school students: The chain mediating role of emotion regulation and psychological resilience. Psychology in the Schools.

[B35-behavsci-16-00894] Li W., Zhao Z., Chen D., Peng Y., Lu Z. (2022). Prevalence and associated factors of depression and anxiety symptoms among college students: A systematic review and meta-analysis. Journal of Child Psychology and Psychiatry.

[B36-behavsci-16-00894] Li Z., Huang K., Ding X., Wang T., Zhou S., Han Y., Chen Z., Wan X., Hao X., Meng Y., Ji Y. (2025). How does mindfulness awareness influence cognitive reappraisal in individuals with mild cognitive impairment? cognitive flexibility as a mediator and age as a moderator. Cognitive Therapy and Research.

[B37-behavsci-16-00894] Lin Z., Cai H., Huang Y., Zhou L., Yuan Z., He L., Li J. (2025). Prevalence of depression among university students in China: A systematic review and meta-analysis. BMC Psychology.

[B38-behavsci-16-00894] Luthans F., Avolio B. J., Avey J. B., Norman S. M. (2007). Positive psychological capital: Measurement and relationship with performance and satisfaction. Personnel Psychology.

[B39-behavsci-16-00894] Niu H., Wang S., Tao Y., Tang Q., Zhang L., Liu X. (2023). The association between online learning, parents’ marital status, and internet addiction among adolescents during the COVID-19 pandemic period: A cross-lagged panel network approach. Journal of Affective Disorders.

[B40-behavsci-16-00894] Paul K., Pourtois G., van Steenbergen H., Gable P., Dreisbach G. (2021). Finding a balance: Modulatory effects of positive affect on attentional and cognitive control. Current Opinion in Behavioral Sciences.

[B41-behavsci-16-00894] Rief W., Joormann J. (2019). Revisiting the cognitive model of depression: The role of expectations. Clinical Psychology in Europe.

[B42-behavsci-16-00894] Scheier M. F., Carver C. S. (1985). Optimism, coping, and health: Assessment and implications of generalized outcome expectancies. Health Psychology.

[B43-behavsci-16-00894] Sousa G. M. d, Lima-Araújo G. L. d., Araújo D. B. d., Sousa M. B. C. d. (2021). Brief mindfulness-based training and mindfulness trait at-tenuate psychological stress in university students: A randomized controlled trial. BMC Psychology.

[B44-behavsci-16-00894] Southward M. W., Holmes A. C., Strunk D. R., Cheavens J. S. (2022). More and better: Reappraisal quality partially explains the effect of reappraisal use on changes in positive and negative affect. Cognitive Therapy and Research.

[B45-behavsci-16-00894] Stanton K., Watson D. (2014). Positive and negative affective dysfunction in psychopathology. Social and Personality Psychology Compass.

[B46-behavsci-16-00894] Troy A. S., Willroth E. C., Shallcross A. J., Giuliani N. R., Gross J. J., Mauss I. B. (2023). Psychological resilience: An affect-regulation framework. Annual Review of Psychology.

[B47-behavsci-16-00894] Ulibarri-Ochoa A., Macía P., Ruiz-de-Alegría B., García-Vivar C., Iraurgi I. (2024). The role of resilience and coping strategies as predictors of well-being in breast cancer patients. European Journal of Oncology Nursing.

[B48-behavsci-16-00894] Walach H., Buchheld N., Buttenmüller V., Kleinknecht N., Schmidt S. (2006). Measuring mindfulness—The Freiburg Mindfulness Inventory (FMI). Personality and Individual Differences.

[B49-behavsci-16-00894] Wang L., Liu H. C., Li Z. Q., Du W. (2007). Reliability and validity of emotion regulation questionnaire Chinese revised version. Chinese Journal of Health Psychology.

[B50-behavsci-16-00894] Wang X., Liu Q. (2022). Prevalence of anxiety symptoms among Chinese university students amid the COVID-19 pandemic: A systematic review and meta-analysis. Heliyon.

[B51-behavsci-16-00894] Watson D., Naragon-Gainey K. (2010). On the specificity of positive emotional dysfunction in psychopathology: Evidence from the mood and anxiety disorders and schizophrenia/schizotypy. Clinical Psychology Review.

[B52-behavsci-16-00894] Wysocki A., McCarthy I., van Bork R., Cramer A. O. J. (2025). Cross-lagged panel networks. Advances.in/Psychology.

[B53-behavsci-16-00894] Yu X., Zhang J. (2007). Factor analysis and psychometric evaluation of the Connor-Davidson Resilience Scale (CD-RISC) with Chinese people. Social Behavior and Personality: An International Journal.

[B54-behavsci-16-00894] Zarotti N., Povah C., Simpson J. (2020). Mindfulness mediates the relationship between cognitive reappraisal and resilience in higher education students. Personality and Individual Differences.

[B55-behavsci-16-00894] Zhang S., Yang R., Cui Y., Zhou Y., Jiang L., Xi J., Fang J. (2025). Negative life events, inadequate mental health literacy, and emotional symptoms among Chinese college students: A school-based longitudinal prospective study. International Journal of Mental Health Systems.

