# Supplementary material for: Longitudinal Relationships Between Positive Psychological Capacities and Emotional Well-Being Among 950 College Students: A Cross-Lagged Panel Network Analysis
Source: Behav Sci (Basel). 2026 Jun 2;16(6):894. doi: 10.3390/bs16060894 (PMC13295284; doi:10.3390/bs16060894)
Supplement: Supplementary file 1 [file behavsci-16-00894-s001.zip › behavsci-4154184-supplementary.pdf]

Supplementary Materials

Longitudinal Relationships Between Positive Psychological Capacities and Emotional Well-Being Among 950 College Students: A Cross-Lagged Panel Network Analysis

Supplementary Table S1. Description and Correlation matrix

Supplementary Table S2. Edge Weight Adjacency Matrix of CLPN

Supplementary Table S3. Baseline Differences Between Completers and Dropouts

Supplementary Figure S1. Bootstrapped 95% Confidence Intervals of the Edge Weights for CLPN

Supplementary Figure S2. Case-Dropping Bootstrapped Centrality Indexes for CLPN

Supplementary Figure S3. Differences in Edge Weight in CLPN

Supplementary Figure S4. Differences in Centrality Indexes in CLPN

Supplementary Figure S5. Cross-Lagged Panel Network with Autoregression Paths

Table. S1 Description and Correlation matrix

	M±*SD*	1	2	3	4	5	6	7	8	9	10	11	12	13	14	15	16	17	18	19	20	21	22	23	24
1.Gender	1.610±0.488	—																							
2.Age	19.236±1.176	-0.052	—																						
3.SES	-1.277±0.410	0.045	-0.195***	—																					
4.Opt1	13.236±2.878	-0.061	-0.01	0.114***	—																				
5.Str1	26.805±4.851	-0.046	-0.025	0.103**	0.727***	—																			
6.Ten1	40.612±8.061	-0.076*	0.013	0.065*	0.683***	0.821***	—																		
7.CR1	28.232±5.804	0.122***	-0.022	0.029	0.373***	0.444***	0.382***	—																	
8.ES1	16.182±4.479	-0.163***	0.015	-0.054*	-0.114***	-0.06	-0.042	0.226***	—																
9.DEP1	10.688±4.163	-0.021	0.024	-0.069*	-0.334***	-0.38***	-0.324***	-0.15***	0.202***	—															
10.ANX1	5.482±2.372	0.01	0.062	-0.06	-0.216***	-0.258***	-0.222***	-0.096**	0.139***	0.717***	—														
11.Awa1	12.027±2.744	0.027	-0.008	0.034	0.435***	0.487***	0.435***	0.381***	-0.049	-0.175***	-0.087**	—													
12.Acc1	16.293±3.735	-0.056	0.025	0.045	0.505***	0.570***	0.573***	0.342***	-0.018	-0.267***	-0.217***	0.594***	—												
13.PA1	19.518±4.272	0.028	-0.054	0.078*	0.438***	0.437***	0.392***	0.287***	-0.129***	-0.417***	-0.348***	0.355***	0.398***	—											
14.NA1	14.368±4.145	0.044	0.009	-0.1**	-0.261***	-0.318***	-0.255***	-0.195***	0.084*	0.51***	0.494***	-0.187***	-0.284***	-0.475***	—										
15.Opt2	13.689±2.687	0.025	0.014	0.012	0.372***	0.324***	0.325***	0.178***	-0.079*	-0.164***	-0.128***	0.245***	0.291***	0.228***	-0.13***	—									
16.Str2	27.652±4.812	0.014	0.058	0.002	0.321***	0.404***	0.388***	0.202***	-0.036	-0.207***	-0.142***	0.264***	0.329***	0.272***	-0.175***	0.74***	—								
17.Ten2	42.492±7.78	0.001	0.045	-0.018	0.316***	0.357***	0.415***	0.189***	-0.055	-0.177***	-0.120***	0.236***	0.308***	0.265***	-0.130***	0.708***	0.843***	—							
18.CR2	28.267±5.520	0.102**	-0.002	-0.036	0.204***	0.241***	0.209***	0.328***	-0.002	-0.123***	-0.067*	0.219***	0.215***	0.157***	-0.099**	0.460***	0.528***	0.495***	—						
19.ES2	15.963±4.350	-0.223***	-0.029	-0.014	-0.092**	-0.045	-0.027	0.044	0.376***	0.076*	0.079*	-0.061	-0.041	-0.076*	0.039	-0.031	0.027	0.045	0.215***	—					
20.DEP2	9.535±3.856	-0.046	-0.047	0.014	-0.171***	-0.162***	-0.151***	-0.085**	0.079*	0.295***	0.262***	-0.077*	-0.089**	-0.164***	0.157***	-0.347***	-0.414***	-0.379***	-0.244***	0.085**	—				
21.ANX2	5.044±2.159	-0.012	-0.049	0.013	-0.141***	-0.137***	-0.131***	-0.044	0.101**	0.276***	0.335***	-0.042	-0.077*	-0.143***	0.16***	-0.296***	-0.341***	-0.335***	-0.167***	0.096**	0.797***	—			
22.Awa2	12.533±2.874	0.043	0.015	-0.004	0.243***	0.242***	0.257***	0.164***	-0.081*	-0.124***	-0.056	0.307***	0.251***	0.206***	-0.103***	0.501***	0.539***	0.533***	0.43***	0.009	-0.227***	-0.19***	—		
23.Acc2	17.471±3.954	-0.022	0.063	-0.028	0.305***	0.322***	0.344***	0.205***	-0.028	-0.196***	-0.144***	0.261***	0.387***	0.271***	-0.151***	0.584***	0.645***	0.674***	0.448***	0.04	-0.329***	-0.306***	0.703***	—	
24.PA2	21.265±4.166	0.02	0.009	-0.028	0.255***	0.23***	0.226***	0.15***	-0.114***	-0.25***	-0.185***	0.162***	0.199***	0.359***	-0.186***	0.482***	0.555***	0.534***	0.356***	-0.049	-0.45***	-0.379***	0.448***	0.551***	—
25.NA2	13.548±3.851	0.099**	-0.064	0.01	-0.157***	-0.201***	-0.171***	-0.137***	0.068*	0.246***	0.234***	-0.116***	-0.174***	-0.234***	0.258***	-0.355***	-0.441***	-0.377***	-0.257***	0.04	0.563***	0.515***	-0.291***	-0.389***	-0.527***

Note: **p*<0.05, ***p*<0.01, ****p*<0.001. Opt1, Opt2 :optimism at T1, T1; Str1, Str2: strength at T1, T2; Ten1, Ten2: tenacity at T1, T2; CR1, CR2: cognitive reappraisal at T1, T2; ES1, ES2: emotion suppression at T1, T2; Awa1, Awa2: awareness at T1, T2; Acc1, Acc2: acceptance at T1, T2; DEP1, DEP2: depression at T1, T2; ANX1, ANX2: anxiety at T1, T2; PA1, PA2: positive affect at T1, T2; NA1, NA2: negative affect at T1, T2

Table. S2 Edge Weight Adjacency Matrix of CLPN

Edge	Edge weight	Edge	Edge weight
PA → Ten	0.153	Awa → Str	0.030
Anx → Dep	0.141	Dep → CR	-0.029
Acc → Ten	0.128	Acc → NA	-0.029
Anx → NA	0.118	Awa → Opt	0.029
Acc → Str	0.112	Opt → Anx	-0.026
Opt → PA	0.097	CR → NA	-0.025
Awa → CR	0.093	Dep → Acc	-0.025
Dep → PA	-0.084	Ten → Opt	0.024
PA → Str	0.081	ES → Awa	-0.021
Opt → Dep	-0.077	Opt → ES	-0.020
Acc → CR	0.073	Dep → Str	-0.020
PA → Acc	0.073	Dep → Anx	0.019
Ten → Str	0.072	PA → Opt	0.017
PA → NA	-0.065	CR → PA	0.016
Ten → Acc	0.058	Acc → Awa	0.015
Str → CR	0.055	ES → NA	0.015
Acc → Opt	0.055	Ten → PA	0.013
Dep → NA	0.054	CR → Acc	0.012
Opt → Acc	0.047	ES → Anx	0.010
ES → CR	-0.044	ES → Opt	-0.010
ES → PA	-0.040	Anx → Opt	-0.004
Opt → Awa	0.035	Opt → Ten	0.002
Str → NA	-0.034	CR → Awa	0.002
Ten → Awa	0.032	Ten → Anx	-0.001
PA → Awa	0.032	Acc → PA	0.001

Table S3. Baseline Differences Between Completers and Dropouts

Baseline Variables	Complete Group (*n*=950)		Dropout Group (*n*=219)		*t*	df	*p*	Cohen's d
	M	*SD*	*M*	*SD*				
AGE	19.236	1.176	19.397	1.189	1.780	304.943	0.076	0.137
SES	-1.277	0.410	-1.340	0.444	-1.875	289.652	0.062	-0.147
Opt	13.236	2.878	13.216	2.969	-0.086	281.31	0.932	-0.007
Str	26.805	4.851	27.057	4.790	0.664	277.921	0.507	0.052
Ten	40.612	8.061	40.655	8.684	0.064	265.214	0.949	0.005
CR	28.232	5.804	27.689	6.041	-1.121	250.965	0.264	-0.092
ES	16.182	4.479	16.289	4.742	0.279	243.311	0.780	0.023
Awa	12.027	2.744	12.011	2.811	-0.074	256.924	0.941	-0.006
Acc	16.293	3.735	16.870	3.908	1.846	251.946	0.066	0.151
DEP	10.688	4.163	11.293	4.915	1.614	259.117	0.108	0.133
ANX	5.482	2.372	5.782	2.638	1.494	274.377	0.136	0.120
PA	19.518	4.272	19.529	4.563	0.032	254.247	0.975	0.003
NA	14.368	4.145	14.563	4.302	0.563	251.340	0.574	0.046

Figure S1 Bootstrapped 95% Confidence Intervals of the Edge Weights for CLPN.

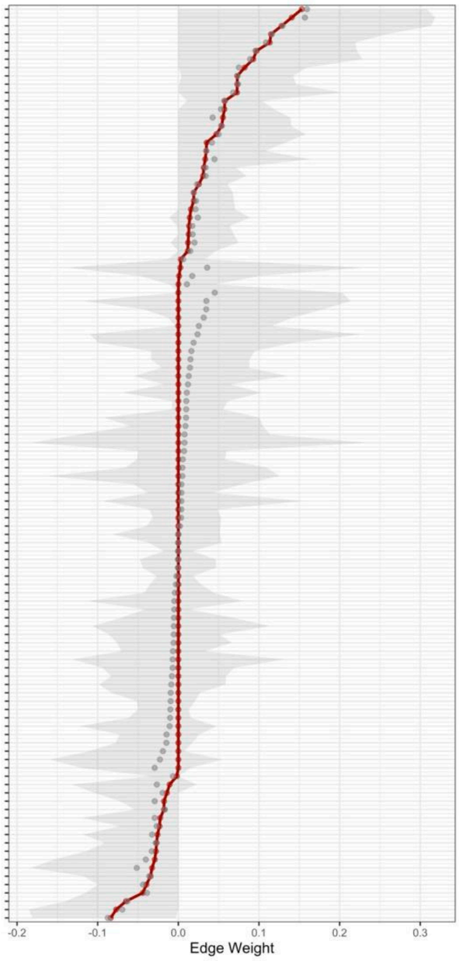

Figure S2 Case-Dropping Bootstrapped Centrality Indexes for CLPN

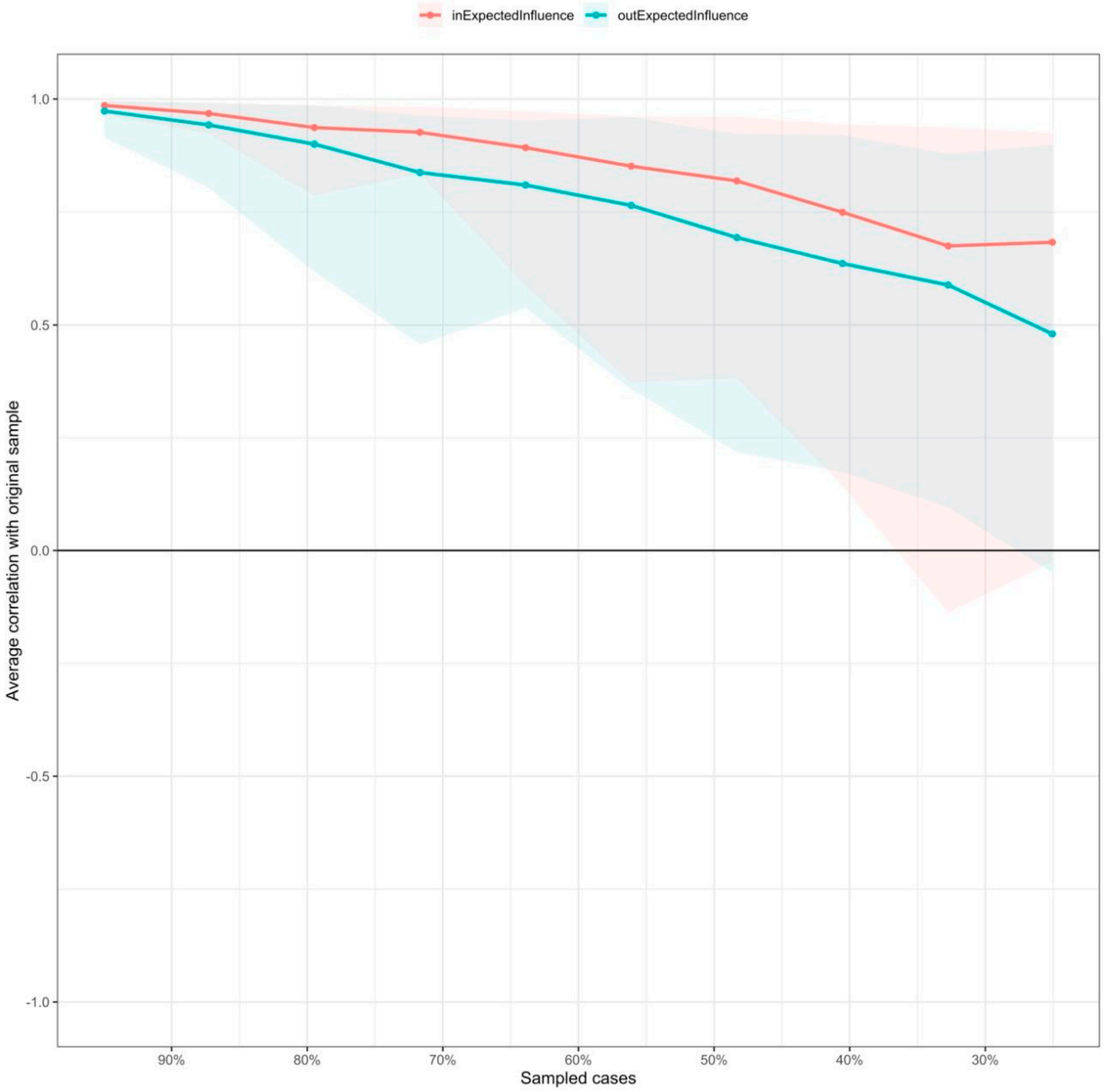

Figure S2 Case-Dropping Bootstrapped Centrality Indexes for CLPN. Lines reflect mean correlations between centrality values of original sample and sub samples with different degrees of persons dropped, and areas around the lines reflect 95% CIs

Figure S3 Differences in Edge Weight in CLPN

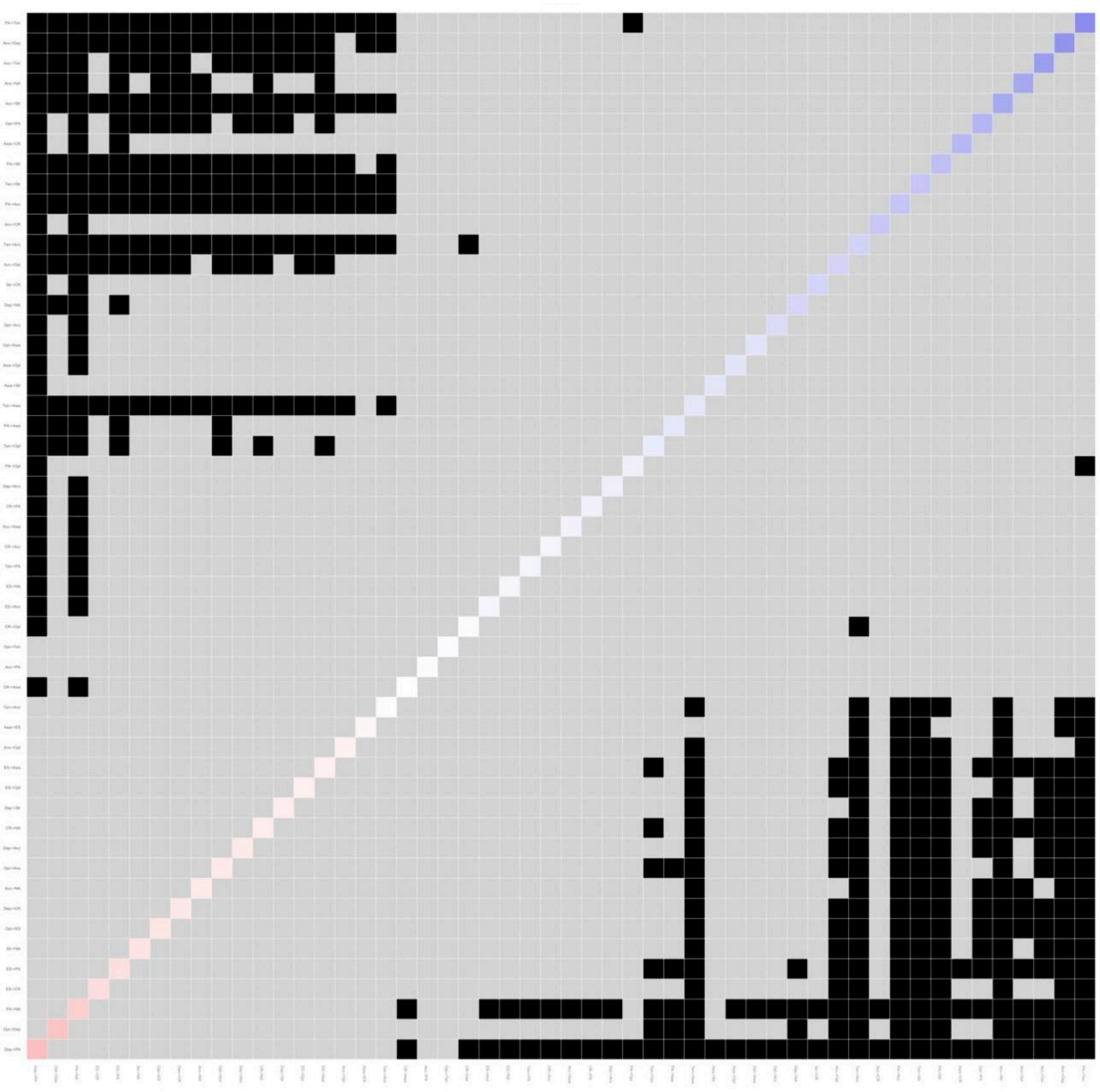

Figure S3 Differences in edge weight in CLPN. Black squares depict a significant difference in strength centrality between two edges and grey squares depict a non-significant difference between two edges

Figure S4 Differences in Centrality Indexes in CLPN

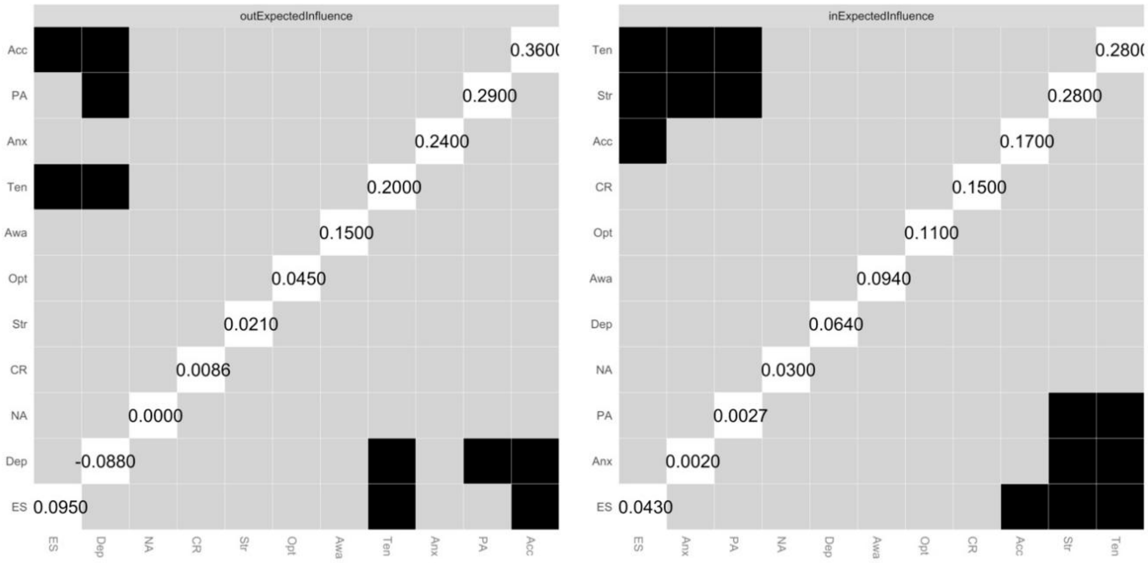

Figure S4 Differences in out-Expected Influence Centrality and in-Expected Influence Centrality in CLPN. Black squares depict a significant difference in strength centrality between two nodes and grey squares depict a non-significant difference between two nodes

Figure S5 Cross-Lagged Panel Network with Autoregression Paths

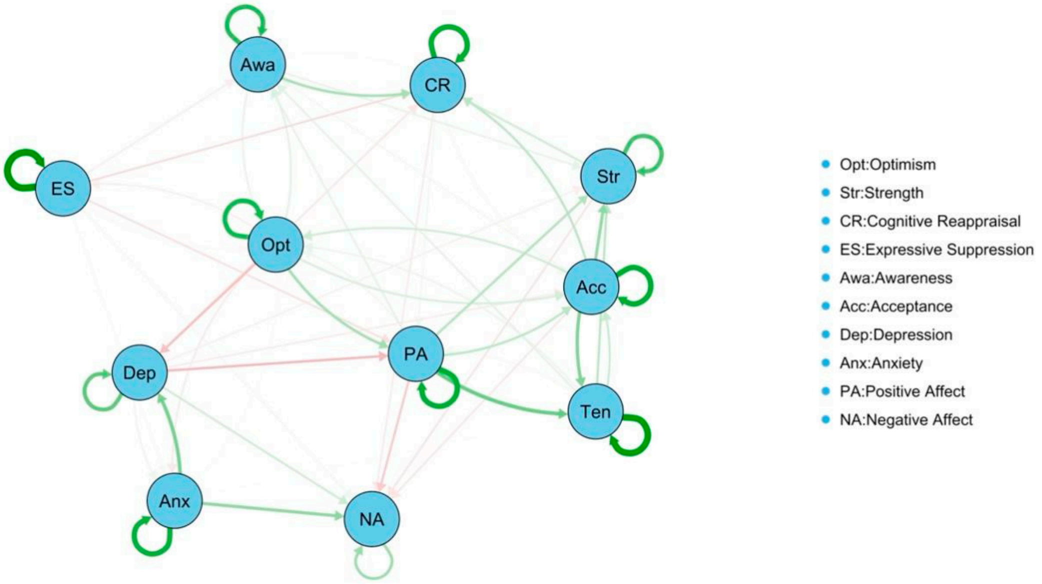

Figure S5 The CLPN with autoregressive paths. Arrows represent longitudinal relationships. Green edges indicate positive relationships, and red edges indicate negative relationships. Thicker edges represent stronger relations. (For interpretation of the references to color in this figure legend, the reader is referred to the web version of this article)
